# Protective Capacity of Monoclonal Antibodies against Acinetobacter baumannii K9 Capsular Polysaccharide

**DOI:** 10.1128/spectrum.04141-22

**Published:** 2023-01-09

**Authors:** Anna Karatovskaya, Natalia Rudenko, Anna Zamyatina, Anton Zvonarev, Vladimir Oleinikov, Anna Shpirt, Andrei Perepelov, Yuriy Knirel, Fedor Brovko

**Affiliations:** a Laboratory of Immunochemistry, Pushchino Branch, Shemyakin-Ovchinnikov Institute of Bioorganic Chemistry, Russian Academy of Sciences, Pushchino, Moscow Region, Russia; b FSBIS FRC Pushchino Scientific Centre of Biological Research, G.K. Skryabin Institute of Biochemistry and Physiology of Microorganisms, Russian Academy of Sciences, Pushchino, Moscow Region, Russia; c Laboratory of Carbohydrates and Biocides, N.D. Zelinsky Institute of Organic Chemistry, Russian Academy of Sciences, Moscow, Russia; d Shemyakin-Ovchinnikov Institute of Bioorganic Chemistry of the Russian Academy of Sciences, Ulitsa Miklukho-Maklaya, Moscow, Russia; Emory University School of Medicine

**Keywords:** *Acinetobacter baumannii*, capsular polysaccharide, monoclonal antibodies, opsonization assay

## Abstract

Acinetobacter baumannii is an antibiotic-resistant opportunistic pathogen, one of the main causes of hospital infections. There is an urgent need for the development of therapy strategies which are not based on antibiotics. Hybridoma technology was used to obtain monoclonal antibodies. The antibodies were characterized by enzyme immunoassay and fluorescence microscopy according to their ability to opsonize A. baumannii and to protect model animals from infection upon intraperitoneal and pulmonary injection. Monoclonal antibodies (MAbs), IgG, against the K9 capsular polysaccharide (CPS) of A. baumannii were prepared using a glycoconjugate, synthesized by squaric-acid chemistry, consisting of two CPS K9 monomer units and a carrier protein. The MAbs were highly specific, stained the bacterial surface, allowed detection of A. baumannii in infected lung tissue, effectively opsonized the bacteria at nanogram concentrations (up to 1.5 ng/mL for CPS-407), and demonstrated a high ability to protect an organism against bacterial infection upon intraperitoneal and lung injection. In intraperitoneal infection of a mouse model with A. baumannii K9, the CPS-407 antibody protected at a dose of 25 μg/mouse. When bacteria were injected into the lung, MAb therapy prevented infection of the body and led to a significant reduction of the bacterial load in infected tissues.

**IMPORTANCE** MAbs detected A. baumannii in infected lung tissue, effectively opsonized bacteria, and protected model animals from infection.

## INTRODUCTION

In recent years, A. baumannii has accounted for a significant proportion of infections in surgical departments (1). The bacterium is widespread and resistant to most antibiotics, which usually leads to increased treatment costs and often to high mortality (2). These facts suggest passive vaccination as an alternative strategy to combat this nosocomial infection (3). Passive vaccination has been successfully used for the prevention and treatment of bacterial infections before the antibiotic era in the form of serum therapy (4). Therapeutic antibodies can stimulate macrophages and the complement system, increasing bacterial clearance and preventing sepsis without affecting the diversity of the host microbiota. There are currently no clinical monoclonal antibodies (MAbs) against A. baumannii infection (5). From both a practical and theoretical point of view, obtaining antibodies against the protective component of the microorganism—the capsular polysaccharide (CPS)—and studying their effect on the development of infection in animal models are of undoubted interest.

Previously, the authors have shown that immunization with conjugates of CPS fragments via protein carriers stimulates and induces protective immune reactions and protects model laboratory animals against infection by A. baumannii (6). In this work, MAbs against the K9 capsular polysaccharide of A. baumannii were prepared by immunization with a glycoconjugate that contained a K9 CPS fragment. The MAbs effectively bound the CPS, detected A. baumannii in infected tissue, and protected model animals against infection. Strain K9 often appears as a cause of bacterial infections (7). Its structure remained stable over a long period of observation (6).

## RESULTS

### Preparation and characterization of A. baumannii K9 anti-CPS MAbs.

To obtain monoclonal antibodies, we immunized mice with a conjugate synthesized by squaric-acid chemistry which included bovine serum albumin (BSA) and a fragment of two oligosaccharide repeats (K-units) of A. baumannii type K9 CPS. As shown earlier, immunization with this conjugate provides a high titer of specific antibodies in blood serum (6), indicating the formation of a sufficient pool of plasma cells to obtain hybridoma cell lines which stably produce MAbs. Splenocytes and cells of the popliteal lymph nodes were used as a source of lymphocytes for hybridomas secreting MAbs against the K9 CPS according to the methods of Kоller and Milstein (8). The selection of hybridomas secreting specific antibodies was performed by indirect enzyme immunoassay (EIA) via the interaction of extracellular supernatants with the K9 CPS immobilized on immunoplates. Based on the evaluation of the proliferative activity and stability of antibody production, we selected four stable hybridoma clones secreting anti-CPS MAbs. All antibodies obtained contained a κ- light chain and a γ1- heavy chain, except for CPS-404-γ2b.

**FIG 1:**
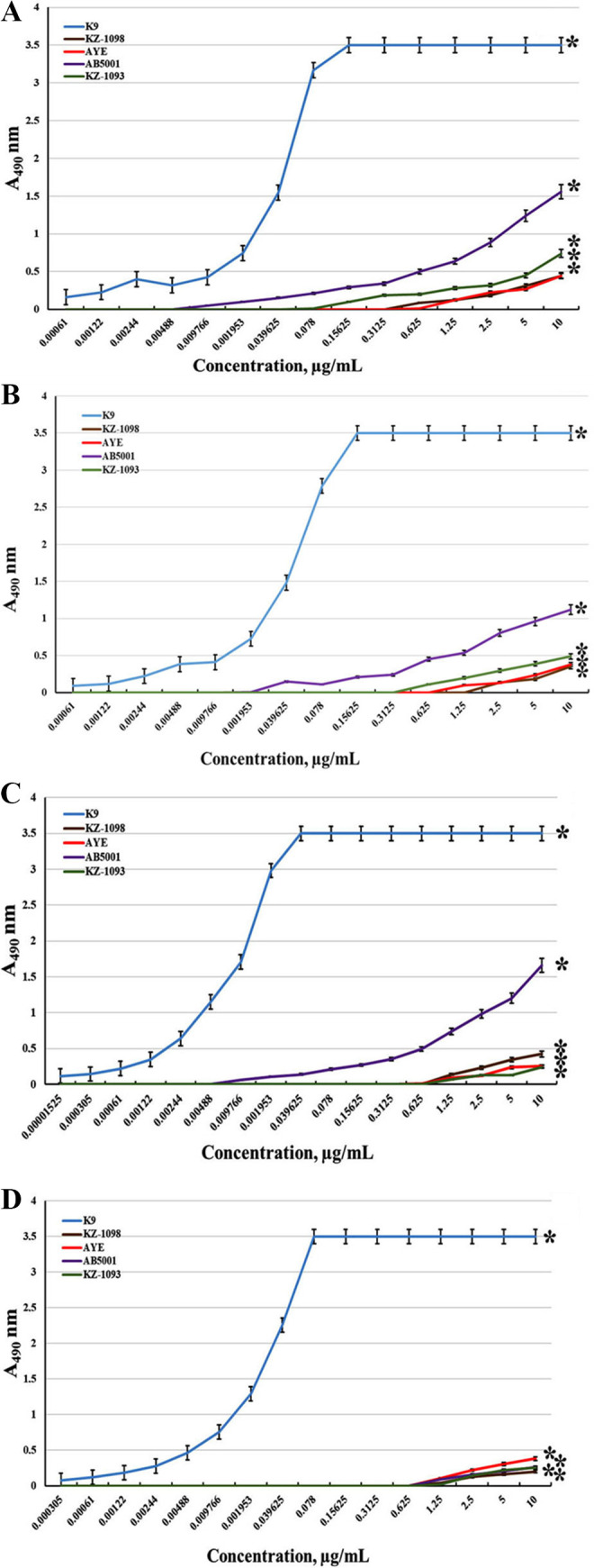
Interaction of monoclonal antibodies (MAbs) CPS-401 (A), CPS-402 (B), CPS-404 (C), and CPS-407 (D) with immobilized Acinetobacter baumannii capsule polysaccharides (CPSs): K9, KZ-1098, AYE, AB5001, and KZ-1093. Data are the means ± standard error of the mean (SEM) of three independent repeats. Asterisk (*) indicates a statistically significant difference (*P* < 0.05, Mann-Whitney test).

All antibodies obtained effectively bound the K9 CPS immobilized on the immunoplate surface. The EIA titration curves are shown in Fig. 1. The antibodies demonstrated high specificity to the K9 CPS, whereas their interaction with CPSs of other A. baumannii strains was insignificant. The CPS structures of the A. baumannii strains used have been shown previously (6).

**FIG 2:**
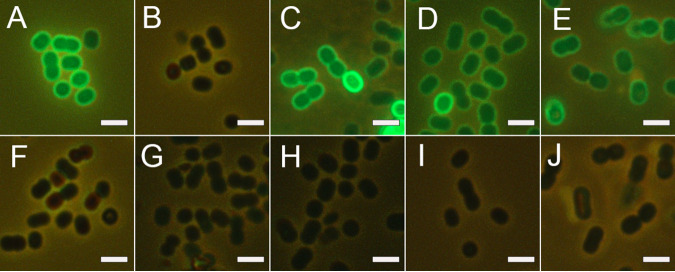
Surface binding of A. baumannii CPS-407–DyLight 488, simultaneous phase contrast and fluorescence microscopy. (A) A. baumannii K9. (B) Isotype control–DyLight 488. (C to E) Clinical strains of A. baumannii K9: MAR 15-2258 (C), MAR 15-4100 (D), and AC54 (E). (F to J) Heterologous strains of A. baumannii: KZ-1093 (F), KZ-1098 (G), AYE (H), AB5001 (I), and K8 (J). Scale bar = 2 μm.

**FIG 3:**
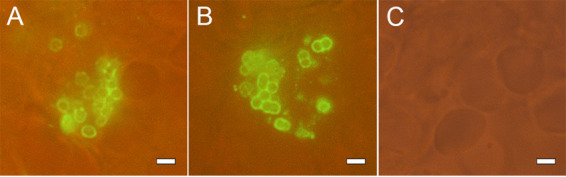
(A and B) Staining of lung tissue of infected mouse with CPS-407–DyLight 488. (C) Isotype control–DyLight 488, simultaneous phase contrast and fluorescence microscopy. Scale bar = 2 μm.

The MAbs obtained effectively stained the surface of bacterial cells, as demonstrated using antibodies marked with DyLight 488. Staining of CPS-407 from A. baumannii K9 is shown as an example in Fig. 2. The antibodies also effectively stained clinical K9 strains and did not stain heterologous strains of A. baumannii (Fig. 2). Using fluorescence-labeled antibodies also enabled the detection of bacteria in the lung tissues of infected animals (Fig. 3).

**FIG 4:**
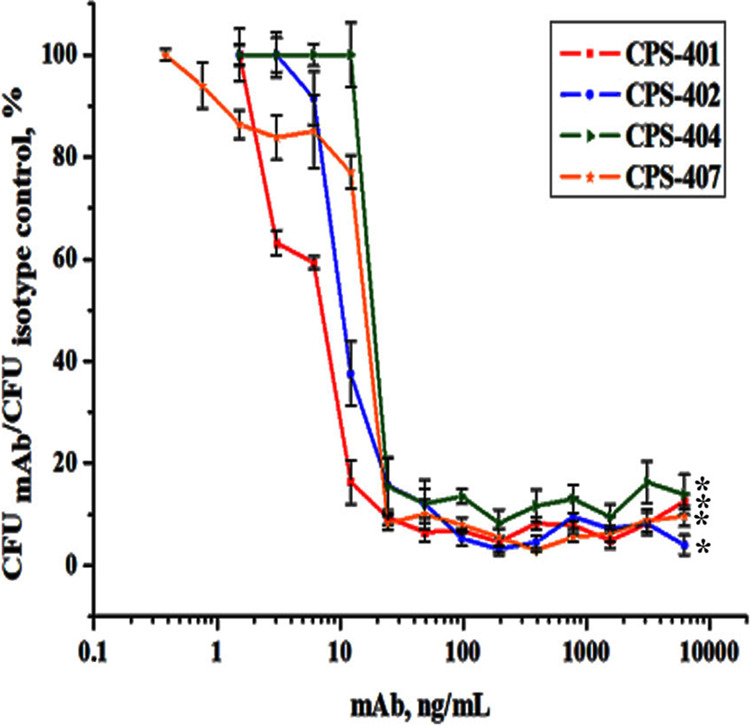
Dependence of the opsonizing capacity of the obtained MAbs on concentration. Data are the means ± SEM of 5 independent repeats. Asterisk (*) indicates a statistically significant difference (*P* < 0.05, Mann-Whitney test).

The antibacterial activity of MAbs was investigated by the opsonization test: opsonization was assessed by decreases in the number of colonies, subtracting the bacteria absorbed by J774 macrophages in the presence of complement system proteins. Opsonization was expressed as a ratio of the number of colonies after incubation with a specific MAb to the number of colonies in the presence of antibodies unrelated to this antigen at the same concentration (isotype control). The CPS-407 antibody, which had the greatest opsonization activity, at a concentration of 1.5 ng/mL, showed a 17% decrease in the number of grown bacteria compared with the control. The graph in Fig. 4 shows the dependence of the opsonization capacity of all obtained antibodies on concentration.

The opsonization capacity of the MAbs relative to other bacterial strains turned out to be less significant compared to the K9 strain (Table 1). This correlated with the data on immunocross activity (Fig. 1). The MAb CPS-407, which had the greatest opsonization capacity relative to the “native” strain, did not opsonize other bacterial strains.

**TABLE 1:** Opsonizing ability of MAbs toward K9 and heterologous strains^*a*^

MAb	Minimum opsonizing concn (ng/mL)				
	K9	AYE	KZ-1098	AB5001	KZ-1093
CPS-401	3 ± 0.03	N/O	N/O	30 ± 0.3	500 ± 5
CPS-402	12 ± 0.12	500 ± 5	N/O	60 ± 0.6	250 ± 2.5
CPS-404	24 ± 0.24	N/O	500 ± 5	30 ± 0.3	N/O
CPS-407	1.5 ± 0.015	500 ± 5	N/O	N/O	N/O

a MAb, monoclonal antibody; CPS, capsule polysaccharide. Data are the means ± standard error of the mean of 5 independent repeats. N/O, no opsonization.

### Survival with CPS-407 when infected with A. baumannii.

**FIG 5:**
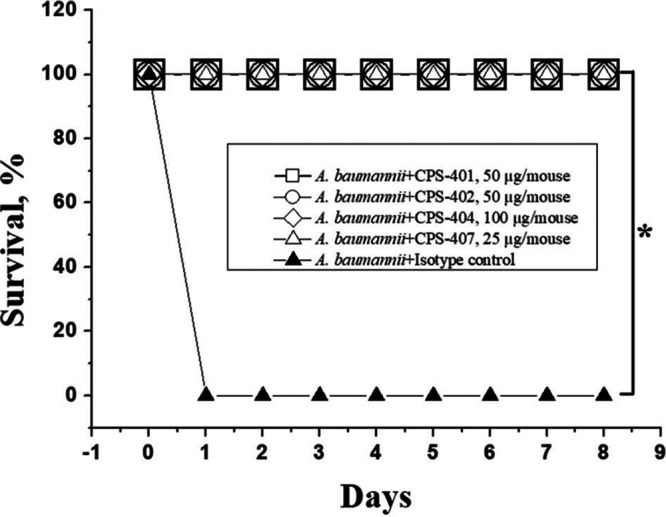
Survival rate of mice infected with intraperitoneal injection of A. baumannii with simultaneous administration of MAbs (*n* = 10 mice/group). An asterisk (*) indicates a statistically significant difference (*P* < 0.05) between the study groups marked with brackets versus the control group that received A. baumannii injections and the control isotype (*n* = 10 mice/group) using a nonparametric logarithmic rank criterion.

The protective ability of MAbs was investigated using two infection models in mice: intraperitoneal and pulmonary administration. In the first case, simultaneous intraperitoneal administration of antibodies and inoculate was used to test the ability of antibodies to protect infected animals. Infection was carried out with a lethal dose of 10^8^ CFU/mouse of A. baumannii K9 (6). The results are shown in Fig. 5. The maximum protective effect was shown by CPS-407, up to a concentration of 25 μg per mouse.

Here, the administration of CPS-407 at a dose of 25 μg/mouse one hour prior to infection prevented the death of the animal. An hour after the infection, the protective dose of CPS-407 increased significantly to 400 μg/mouse.

**FIG 6:**
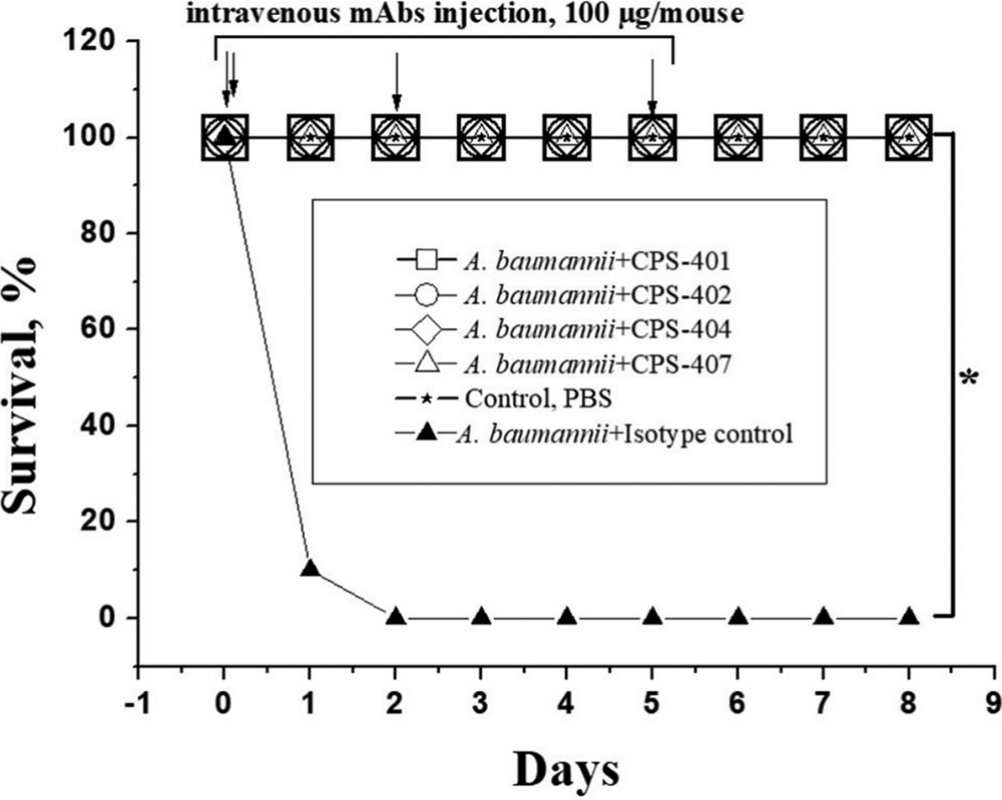
Survival of mice challenged with A. baumannii by lung injection using CPS-407 therapy (*n* = 10 mice/group). An asterisk (*) displays a statistically significant difference between study groups marked with brackets versus the control group that received A. baumannii injections and the control isotype (*n* = 10 mice/group) by the nonparametric log rank test (*P* < 0.05).

The protective capacity of the antibodies was also tested in the case of infection by an injection of the inoculate directly into the lung. In comparison with intranasal and intratracheal infection models, the pulmonary model made it possible to bypass the mechanical and nonspecific barriers to infection, as well as the components of innate immunity that are active in the respiratory tract. Bacteria were injected in a minimal volume (10 μL), which resulted in the creation of a lesion directly in the lung without injuring the animal; control animals injected with phosphate-buffered saline (PBS) alone did not show any signs of discomfort or disease. The use of the pulmonary infection model made it possible to significantly (by a factor of five) reduce the lethal dose of infection. The lethal dose with this administration route was 2 × 10^7^ CFU/mouse of A. baumannii K9. The MAbs were administered intravenously four times: simultaneously with infection and after 2, 48, and 120 h. All animals injected with each therapeutic antibody survived and showed no signs of discomfort during the entire follow-up period compared to those without antibody therapy (only nonessential MAbs; isotype control), which died. Figure 6 shows the results of an experiment with MAbs; the data were the same for all antibodies.

**FIG 7:**
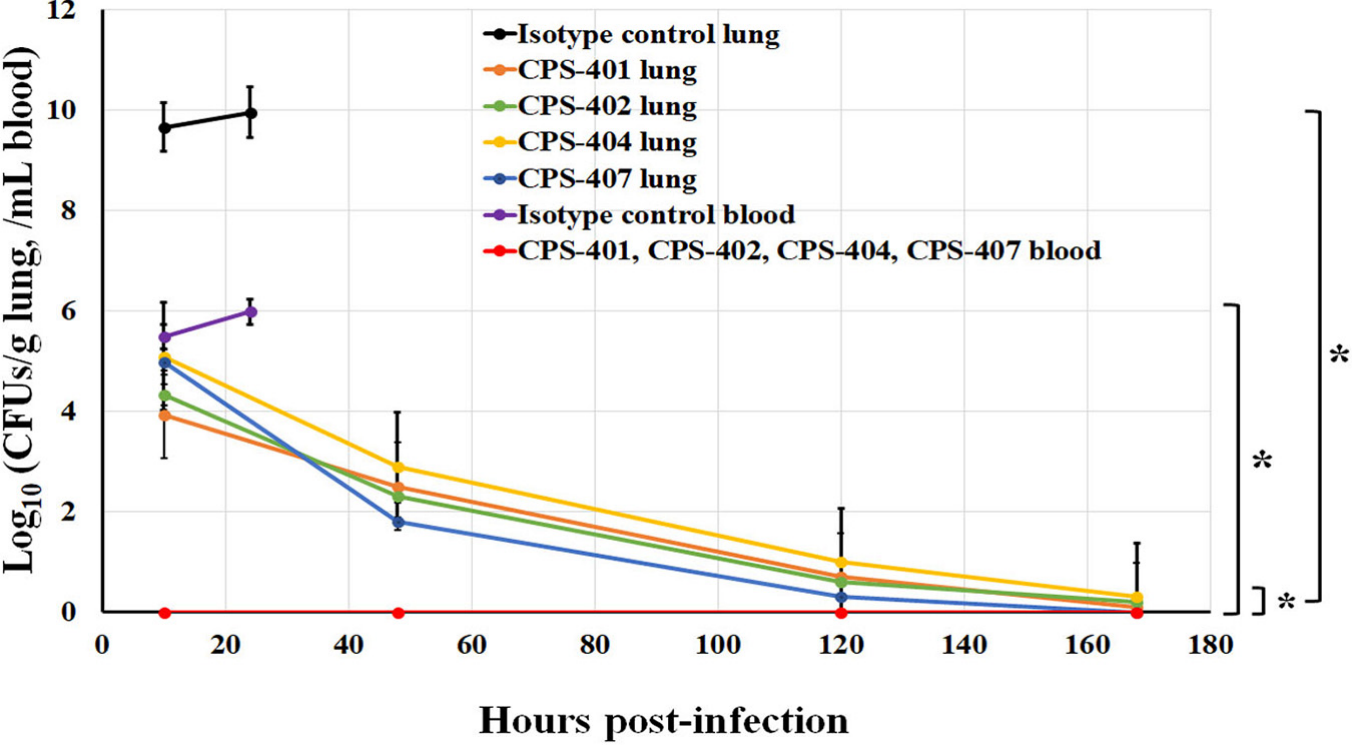
Bacterial load was evaluated in the lungs and blood of mice challenged with A. baumannii by lung injection at 10, 48, 120, and 168 h postinfection, *n* = 5 mice/group. Data are the means ± SEM of 5 independent repeats. The asterisk (*) shows statistically significant differences in CFU (*P* < 0.05, Mann-Whitney test) between study groups marked with brackets.

In the pulmonary model, analysis of the presence of A. baumannii in the blood and lungs showed that the introduction of antibodies into the caudal vein completely prevented bacteria from entering the blood; bacteria were not detected in the blood during the entire observation period. The introduction of antibodies to A. baumannii CPS into the bloodstream of animals led to a noticeable decrease in bacterial load in the lung tissue. After the fourth injection, the use of MAb CPS-407 led to complete elimination of infection from the lungs (Fig. 7).

## DISCUSSION

Monoclonal antibodies are a promising tool for post-exposure prophylaxis of infections caused by polyresistant strains of A. baumannii. The search for candidate antigens for passive therapy can be facilitated by the integration of fundamental knowledge in such fields as genomics, proteomics, metabolomics, transcriptomics, and others (9). To date, many protein antigens have already been tested. Examples include the therapeutic potential of MAbs against the biofilm-stabilizing DNABII protein for the treatment of biofilm-related infections in combination with standard antibiotics (10); the Hyr1 protein, which is homologous to the cell surface proteins of Gram-negative bacteria (11).

Capsular polysaccharides help bacteria to survive in host tissues during infection (12), induce an immune response independent of T cells, and are incapable of inducing memory cells unless they are conjugated to protein (13, 14).

A promising antigen for the development of passive immunization (13, 15) is poly-*N*-acetyl-b-(1,6)-glucosamine, a surface exopolysaccharide important for maintaining the integrity of biofilms and protecting A. baumannii from innate host defenses (16); however, this target is not only present in the pathogen.

In this study, a glycoconjugate synthesized by squaric-acid chemistry was used to obtain monoclonal antibodies against the capsular polysaccharide. The advantage of this conjugation method was described previously (6). The obtained MAbs effectively protected animals infected with A. baumannii K9 by various infection methods and, being administered into the lung tissue, they did not allow the bacteria to spread through the body (bacteria were absent in the blood).

OmpA and possibly other A. baumannii outer membrane proteins are masked by the capsular polysaccharide and therefore cannot be a target for passive immunization (17).

The broad variability of capsular polysaccharides, expressed in a large variety of bacterial strains, limits the use of antibodies directed against them (18). The antibodies obtained in this work were specific to the original A. baumannii strain and had practically no cross-reactivity with CPS from other strains. Apparently, the future lies in the use of less specific and more cross-reactive antibodies; the use of multivalent antibody-based drugs aimed at several targets is also possible. It should be noted that A. baumannii type K9 is one of the most widespread clinical isolates of A. baumannii (7). It is important that the antibodies obtained in this work are directed against the СPS of the strain isolated in various hospitals, with a structure that is stable over a long period of time (19).

## MATERIALS AND METHODS

### Production and isolation of MAbs.

Animals used for all experiments conducted in this study, including the challenge with A. baumannii, were performed according to protocol registration no. 852/21 of 10.12.2021, approved at a meeting of the Institute’s Animal Care and Use Committee (BIBCh RAS) on 17 December 2021. Animals were obtained from the Laboratory Animal Breeding Nursery, Pushchino Branch, Institute of Bioorganic Chemistry, Russian Academy of Sciences, which has earned international AAALACi accreditation.

Immunization of 2- to 3 -month-old female BALB/c mice with a conjugate consisting of BSA and two monomer units was carried out as previously described (6) plus two boosters at 1-month intervals. Seven days after the last injection, blood was taken from the caudal vein, and the content of specific antibodies was assessed by indirect EIA in order to choose an animal with a higher titer. Three days before cell fusion, 50 μg of conjugate in PBS was injected subcutaneously into each animal’s paws and intraperitoneally. Hybridomas secreting MAbs were obtained by hybridoma technology (8) with modifications (20).

MAbs were isolated by affinity chromatography on Protein A Sepharose (Thermo Fisher Scientific, USA) (21) from hybridoma culture fluids secreting these MAbs.

The types of heavy and light immunoglobulin chains were determined by EIA using the Rapid ELISA Mouse MAb Isotyping kit (Thermo Fisher Scientific, USA) according to the manufacturer’s instructions.

### Indirect solid-phase enzyme immunoassay.

Purified A. baumannii CPSs were adsorbed on the surface of wells of high-binding EIA immune plates (Corning Costar 2481, Corning Life Sciences, USA) from a 1 μg · mL^−1^ solution in 0.05 М carbonate buffer (рН 9.6) (50 μL). The adsorption was continued overnight at 4°C. The free binding sites of plastic were blocked by incubation with PBS containing 0.1% Tween 20 (PBST) for 1 h, and the samples to be examined (supracellular supernatants, immune sera, purified MAbs) were introduced into the plate wells. If necessary, the samples were prediluted with PBST. Incubation with the immobilized polysaccharides was performed at 37°C for 1 h. Next, the immune plate wells were washed with PBST no less than 6 times, and the conjugate of antibodies against mouse immunoglobulins was added (Corning, Thermo Fisher Scientific 31432, Goat anti-Mouse IgG [H+L] horseradish peroxidase conjugate) in diluted PBST as indicated by the manufacturer and incubated at 37°C for 1 h. A 4-mM solution of an ortho-phenylenediamine peroxidase substrate (Sigma-Aldrich, USA) in citrate phosphate buffer (26 mM citric acid, 50 mM Na_2_HPO_4_ [рН 5.0]) containing 0.003% (vol/vol) Н_2_O_2_ was added. After the development of color, the reaction was stopped by adding an equal volume of 10% (vol/vol) sulfuric acid, and optical absorption was measured at 490 nm using an iMark microplate reader (Bio-Rad, USA).

### Fluorescence microscopy.

MAbs were conjugated with DyLight 488 (cat no. 53025, Thermo Fisher Scientific, USA) according to the manufacturer’s instructions (DyLight Microscale Antibody Labeling Kits, Thermo Fisher Scientific).

For fluorescence microscopy, A. baumannii in the logarithmic growth stage was washed free from the 2YT medium (16 g · L^−1^ bactotryptone, 1 g g · L^−1^ yeast extract, 5 g · L^−1^ NaCl [pH 7.0]) by centrifugation at 1,500 × *g* for 10 min at +4°C. The bacterial precipitate was resuspended in a 1% gelatin solution in PBST and incubated for 15 min at room temperature. The suspension of A. baumannii cells was recentrifuged, and a mixture of MAbs and DyLight 488 (5 μg/mL) in PBST was added to the bacterial sediment and incubated with gentle stirring for 1 h at 37°C in the dark. Additionally, bacterial cells were washed twice by centrifugation in 20 mM HEPES, 0.15 M NaCl, and 0.1% Tween 20; the final washing was carried out in 20 mM HEPES, 0.15 M NaCl.

Mice were infected with A. baumannii by injection of 10^7^ CFU/mouse into the lung. At 24 h after injection, the lung was removed and placed in a petri dish on a filter soaked in a solution containing 2% formaldehyde and 0.5% glutaraldehyde. Next, it was incubated without air access for 48 h at +4°C. To obtain fluorescent micrographs, thin slices were made using a razor blade. The slices were successively incubated in a 1% gelatin solution in PBST (30 min, room temperature), 5 μg/mL MAb-DyLight 488, and PBST with gentle stirring for 1 h at 37°C in the dark. Nonspecific binding was washed free 5 times with PBST with gentle stirring and twice in 20 mM HEPES and 0.15 M NaCl. Lung tissues prepared in this way were used for fluorescence microscopy.

An Axio Imager A1 (Zeiss, Germany) was used to acquire images. The excitation filters BP470/27 were placed in the DG5 filter box. The dichroic cube holds the beam splitter (DBS 490 + 575) and dual bandpass filter (DBP 512/30 + 630/98) from DyLight 488 with excitation 493 nm and emission 518 nm. Microscopy was carried out in a combined mode with simultaneous phase contrast and fluorescence.

### Opsonization assay.

The assay was carried out as described previously (6); dilutions of the obtained MAbs were used, and nonessential MAbs were used as a negative (isotype) control.

### The protective ability of MAbs in infection with A. baumannii K9.

The protective capacity of the antibodies obtained was tested with two infection methods: intraperitoneal (antibodies were also injected intraperitoneally) and injection into the lung (antibodies were injected intravenously into the caudal vein). Male BALB/c mice, 5 to 6 months old, were used for infection.

For infection, the strain A. baumannii K9 was cultivated overnight at 37°C under stirring in 2YT medium, and then cultivated under the same conditions until the middle of the logarithmic growth phase. The concentration of A. baumannii was confirmed by quantitative cultivation in a mixture of 2YT medium and 1.5% agar. The bacteria were washed twice with PBS by centrifugation at 1,500 × *g.* In the intraperitoneal model, the suspension of bacteria in PBS (lethal dose: 10^8^ CFU per mouse) was injected at a volume of 200 μL, and MAbs at a volume of 100 μL.

In the pulmonary model, the inoculate was injected into the lung at a volume of 10 μL (lethal dose: 2 × 10^7^ CFU per mouse) in PBS from the dorsal side of the animal under the lower rib at a distance of 0.5 cm from the spine (31G needle). During the development of this method and in further experiments, PBS was administered as a control injection. MAbs were injected intravenously at a volume of 200 μL.

### Statistical analysis.

The analysis was performed using Microsoft Excel and Origin Pro 8 software. Statistical analysis was based on the Shapiro-Wilk normality test. An unpaired Mann-Whitney test was used to analyze statistical significance between experimental groups. A nonparametric log-rank test was used in the survival analysis. Significance was achieved at *P* < 0.05. Data are resented as means ± standard error of the mean (SEM).
